# Assessing next-generation sequencing-based computational methods for predicting transcriptional regulators with query gene sets

**DOI:** 10.1093/bib/bbae366

**Published:** 2024-07-31

**Authors:** Zeyu Lu, Xue Xiao, Qiang Zheng, Xinlei Wang, Lin Xu

**Affiliations:** Department of Statistics and Data Science, Moody School of Graduate and Advanced Studies, Southern Methodist University, 3225 Daniel Ave., P.O. Box 750332, Dallas, TX, United States; Quantitative Biomedical Research Center, Peter O’Donnell Jr. School of Public Health, University of Texas Southwestern Medical Center, 5323 Harry Hines Blvd, Dallas, TX, United States; Division of Data Science, College of Science, University of Texas at Arlington, 501 S. Nedderman Dr., Arlington, TX 76019, United States; Division of Data Science, College of Science, University of Texas at Arlington, 501 S. Nedderman Dr., Arlington, TX 76019, United States; Department of Mathematics, University of Texas at Arlington, 411 S. Nedderman Dr., Arlington, TX 76019, United States; Quantitative Biomedical Research Center, Peter O’Donnell Jr. School of Public Health, University of Texas Southwestern Medical Center, 5323 Harry Hines Blvd, Dallas, TX, United States; Department of Pediatrics, Division of Hematology/Oncology, University of Texas Southwestern Medical Center, 5323 Harry Hines Blvd., Dallas, TX, United States

**Keywords:** transcriptional regulator, benchmarking, query gene set, next-generation sequencing, prediction

## Abstract

This article provides an in-depth review of computational methods for predicting transcriptional regulators (TRs) with query gene sets. Identification of TRs is of utmost importance in many biological applications, including but not limited to elucidating biological development mechanisms, identifying key disease genes, and predicting therapeutic targets. Various computational methods based on next-generation sequencing (NGS) data have been developed in the past decade, yet no systematic evaluation of NGS-based methods has been offered. We classified these methods into two categories based on shared characteristics, namely library-based and region-based methods. We further conducted benchmark studies to evaluate the accuracy, sensitivity, coverage, and usability of NGS-based methods with molecular experimental datasets. Results show that BART, ChIP-Atlas, and Lisa have relatively better performance. Besides, we point out the limitations of NGS-based methods and explore potential directions for further improvement.

## Introduction

In the 1960s, foundational principles of gene transcriptional regulation were established through seminal work [1] and subsequent studies, which highlighted the pivotal role of transcriptional regulators (TRs) in controlling the transcription of hundreds or even thousands of genes [2, 3]. TRs are proteins encoded by genes, and their regulation of transcription directly influences the expression levels of genes, playing a crucial role in various cellular processes including cell growth, differentiation, morphogenesis, and death [4–6]. A significant portion of these TRs are transcription factors (TFs), which bind to specific DNA sequences. The remainder of TRs consists of cofactors recruited by TFs and chromatin regulators that can alter the structure and function of chromatin [7, 8].

Evidence has linked multiple known diseases to the dysfunction of specific TRs [9, 10], highlighting the importance of identifying functional TRs in biological and medical research, which can aid the discovery of biomarker genes or potential therapeutic targets [11–13]. However, accurately determining the interactions between TRs and their targets requires significant computational and experimental efforts. Amid these challenges, one intriguing question that draws many researchers’ attention is how to accurately predict the TRs that regulate a user-provided gene set that can be derived from sources such as differentially expressed gene (DEG) analysis, gene ontology analysis, and pathway enrichment analysis.

In the past, a variety of computational approaches have been proposed to answer the aforementioned question based on microarrays and sequencing technologies. Among them, motif enrichment analysis was initially developed by associating over-represented transcription factor binding motifs (TFBMs) with cis-regulatory elements (CREs) (e.g., promoters and enhancers), which regulate the transcription of nearby genes [14]. TFBMs are short DNA subsequences that have high affinities for specific TFs. If the TFBMs are over-represented, the related TFs are claimed to be functional regulators. Many methods have been developed following this rationale [15–17]. However, motif-based methods lack the ability of capturing context-specific binding profile. These methods rely on pre-defined motif libraries such as JASPAR and HOCOMOCO to scan the genome for potential binding sites [18, 19]. The high similarity between TFBMs of distinct TFs and the lack of concrete binding motifs for co-factors can hinder performance [20, 21]. Besides, TFBMs cannot capture the cooperative binding of TFs [22], or the interaction between binding sites and chromatin accessibility/epigenetic modifications, leading to high false-positive rates. Thus, motif enrichment analysis is not sufficient for accurately identifying TRs.

We further mention that the identification of TRs can serve as a downstream application of network construction analysis as well [23–25]. The results from gene regulatory network (GRN) reconstruction methods can be used to cross-validate and enhance the performance of NGS-based approaches. GRN methods can pinpoint key TRs and central genes within the network, providing valuable insights into their regulatory roles. By comparing these key TRs with those predicted by NGS-based approaches, researchers can cross-validate findings and improve prediction accuracy. Furthermore, the TR–gene interactions identified through GRN reconstruction can refine and validate target gene (TG) lists, reducing the risk of false associations based solely on NGS data. However, GRN reconstruction is a complex field with its own extensive literature and methodologies [26–28]. As such, a comprehensive review of these methods is beyond the scope of this current study.

This review focuses on computational methods that can leverage recent advancement of next-generation sequencing (NGS) techniques, especially epigenomic techniques (e.g., ChIP-seq, ATAC-seq, and DNase-seq) to identify TRs that regulate a user-provided gene set. These methods are referred to as NGS-based method in this review, which typically leverage the following types of data.

Chromatin immunoprecipitation followed by sequencing (ChIP-seq) including TR ChIP-seq and Histone ChIP-seq is a widely accepted technique to examine genome-wide DNA–protein interactions. TR ChIP-seq datasets serve as the foundation of all NGS-based methods. A TR ChIP-seq dataset can accurately record the binding sites of one-specific TR [29], and therefore makes it possible to establish the TR–gene connection.

Additionally, histone ChIP-seq techniques (e.g., H3K27ac ChIP-seq) and chromatin accessibility profiling techniques (e.g., DNase-seq, FAIRE-seq, and ATAC-seq) can measure genome-wide epigenetic modification sites or accessible chromatin regions [30, 31]. Unlike TR ChIP-seq data required by all methods, data generated by these techniques cannot be directly associated with one specific TR, and so are only included by some methods for improving the prediction accuracy.

Unlike motif-based methods, which rely on computationally predicted binding motifs, NGS-based approaches leverage experimental data (e.g., ChIP-seq, ATAC-seq) to directly capture the context-specific binding profiles of TRs *in vivo*. By identifying actual binding sites and their surrounding epigenetic context, these methods provide a more accurate and comprehensive understanding of TR activity within specific cellular processes.

The interesting features extracted from these data are commonly termed as ‘peaks’ [32], which are short epigenomic regions that show a significant enrichment of epigenetic marks or factors compared to the background levels. Peaks can indicate different features depending on the NGS techniques, such as TR binding sites (TR ChIP-seq), accessible chromatin regions (ATAC-seq, DNase-seq, FAIRE-seq), or sites of active promoters and enhancers (H3K27ac ChIP-seq).

**Figure 1:**
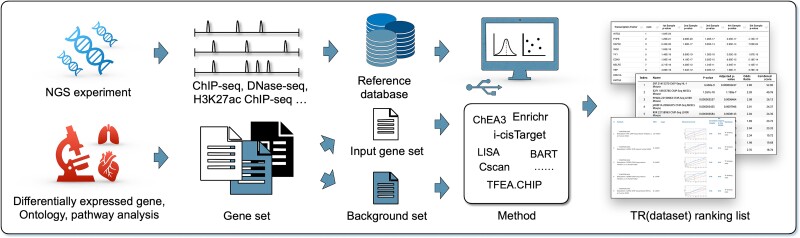
A general workflow of computational methods for predicting transcriptional regulators. Query gene set is produced by DEG analysis, ontology, and pathway analysis. The reference database consists of NGS data from TR ChIP-seq, DNase-seq, and H3K27ac ChIP-seq. Eleven NGS-based computational methods so far have been developed to identify TRs. The final output is typically a TR ranking list.

Until now, over 10 computational methods have been developed to predict TRs using NGS data. These methods are Cscan [33], ENCODE ChIP-seq significance tool [34], Enrichr [35], i-cisTarget [36], RegulatorTrail [37], BART [38], ChIP-Atlas [39], TFEA.ChIP [40], ChEA3 [41], MAGIC [42], and Lisa [43]. Figure 1 illustrates a common workflow of these methods. The input is typically a query set of genes provided by a user. The background set by default contains all annotated genes but also can be provided by the user. The reference database, composed of NGS data, functions as a unique ‘fingerprint’ repository for TRs and genes. Each TR ChIP-seq dataset in the database represents a distinct TR ‘fingerprint’. Users can compare a ‘fingerprint’ from a new cellular study against ‘fingerprints’ in this database and identify functional TRs based on their similarity. The methods used integrate input, background, and reference data to produce a TR ranking list, where higher ranks indicate a greater likelihood of the TR regulating the input gene set. In this sense, the methods discussed in this review mainly act as a ranking algorithm to rank TRs covered by their internal database based on the user input.

Although each of these methods has been shown to perform well in certain applications, a unified and systematic evaluation has not been conducted yet. Existing reviews primarily offer broad overviews of applications in this field, lacking in-depth discussion of individual method details and objective quantitative benchmarking [44, 45]. Considering their extensive use in thousands of publications so far [46–49], it is essential to elucidate their strengths and limitations by a benchmark study to avoid misinterpretation and inform the development of improved methods. The contribution of this review is three-fold. First, it offers a detailed overview of the NGS-based computational methods, highlighting their differences and similarities. Second, it provides an objective evaluation using an extensive collection of gene sets derived from TR perturbation experiments. Third, it systematically assesses key aspects of the methods, including accuracy, sensitivity, coverage, and usability, as well as limitations and directions for potential improvement.

## Categorization of NGS-based methods

**Figure 2:**
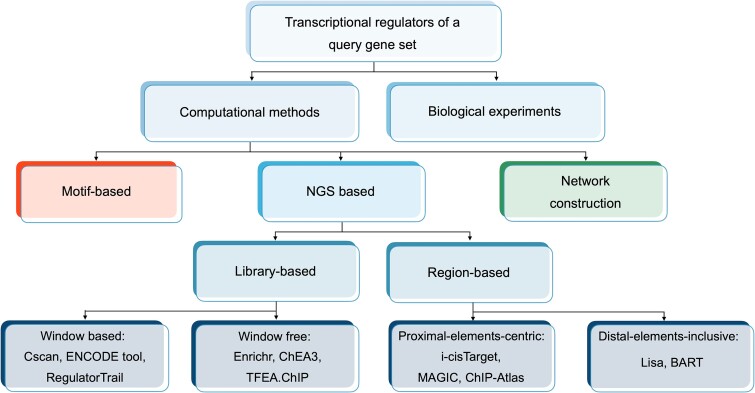
Categorization of computational methods for prediction of TRs mainly based on their approaches.

The NGS-based methods reviewed in this article fall into two main categories, each with two sub-categories: (i) library-based methods, relying on enrichment analysis of TG libraries, subdivided into window-based and window-free approaches based on the main TG assignment algorithm, and (ii) region-based methods, which use cis-regulatory regions (CRRs) for TR prediction, further categorized into proximal-elements-centric and distal-elements-inclusive methods (Fig. 2).

### TRs with multiple ChIP-seq datasets

Due to the uneven allocation of research resources across different TRs, while a TR ChIP-seq dataset is only linked to one specific TR, a single TR may be associated with multiple ChIP-seq datasets in an epigenomic database used. Methods including Cscan, Enrichr, ENCODE ChIP-seq significance tool (ENCODE tool), i-cisTarget, Lisa, and ChIP-Atlas generate a rank for each of the ChIP-seq datasets, even when they are linked to the same TR. Therefore, a final ranking list from any of these methods can contain multiple ranks for any TR with multiple ChIP-seq datasets. In contrast, the rest methods have an integration step to assign one unique rank to each TR. This integration can be achieved through various approaches, such as calculating the mean rank, selecting the highest rank, or employing statistical tests.

### Library-based methods

Predicting TRs that regulate a specific gene set requires establishing a link between TR binding sites and input gene symbols. Two main approaches exist: (i) converting TR binding sites to TG sets followed by enrichment analyses to determine whether each target set is enriched by the input genes compared to the background set of genes and (ii) converting input gene symbols to their regulatory regions and identifying TRs with the highest proportion of binding peaks within these regions. Crucial to both strategies are the gene transcription start site (TSS), which serves as a reference point for mapping gene symbols to chromatin locations. This allows the calculation of the linear epigenomic distance between a TR binding peak and the TSS, a key measure for quantifying the TR–gene connection.

**Figure 3:**
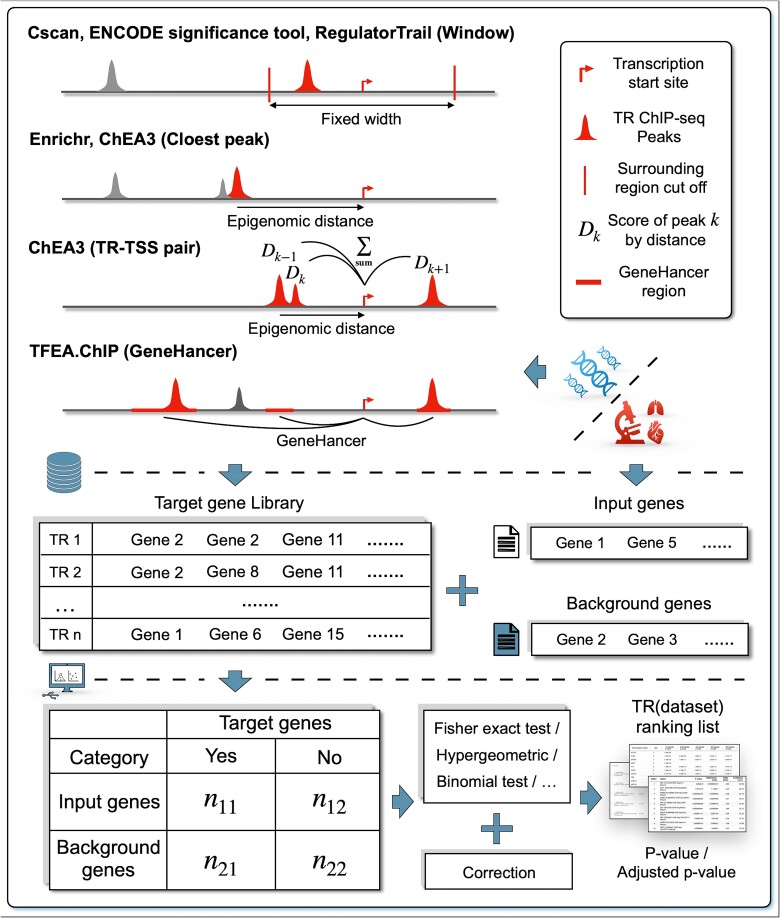
The common workflow of library-based methods. To set up a target gene library, TR ChIP-seq peaks are associated with the nearest genes, usually by measuring the genomic distance linearly to the TSS or whether they fall into the surrounding region. The library can also be derived from experimental evidence. Fisher exact test is commonly used to decide whether the target gene set corresponding to each TR or dataset in the library is enriched with the input genes. The final output is a TR (dataset) ranking list based on the test significance.

Library-based methods, including Cscan, ENCODE ChIP-seq significance tool, Enrichr, RegulatorTrail, ChEA3, and TFEA.ChIP, follow the first approach to create a TG library for TRs or TR datasets (Fig. 3), where the library contains TG sets identified from individual TR ChIP-seq experiments (Cscan, ENCODE tool, Enrichr, ChEA3) or integrated from multiple experiments for the same TR (RegulatorTrail, TFEA.ChIP). Genes in the input and background sets are dichotomized according to each TG set in the library, and a statistical test, usually the Fisher exact test, is then applied to compute the statistical significance of each TG set enriched with the input genes. Each TR or dataset is then ranked based on the statistical significance of their associated TG set.

Library-based methods mainly differ in their TG assignment algorithms. Cscan, ENCODE tool, and RegulatorTrail, for example, employ a ‘window-based’ approach. They define a fixed window (typically 50 bp–10 kb) around each gene’s TSS. If a TR ChIP-seq dataset has peaks within this window for a particular gene, that gene is then classified as a TG.

The other three methods mostly use a ‘window-free’ approach. For example, Enrichr and ChEA3 focus on the proximity of peaks to genes. These methods sort peaks in a TR ChIP-seq dataset based on their linear epigenomic distances to the TSS of nearby genes and retain a certain number of genes with the closest peaks as targets. ChEA3 can further consider the density of peaks by employing a different method: first calculate a score for each TR-TSS pair by aggregating all the peaks of a TR ChIP-seq dataset within a specific range based on their distance to the TSS. Genes associated with TR-TSS pairs having the top 5% highest non-zero scores in each dataset are then designated as targets.

TFEA.ChIP is another ‘window-free’ method, using the GeneHancer database that stores CREs of genes inferred from multiple data sources [50], such as FANTOM5 eRNA-gene expression correlation. These CREs are defined as ‘elite’ regions, and TFEA.ChIP assigns the TGs of a TR based on whether any ChIP-seq dataset of the TR has peaks in these ‘elite’ regions.

There are three key limitations to these six library-based methods. First, these methods use hard cutoffs to identify TGs. This binary approach oversimplifies the complexity of gene regulation. In Cscan, ENCODE ChIP-seq significance tool, and RegulatorTrail, the cutoff determines the window size, and any peaks outside the window are excluded from consideration. Similarly, Enrichr and ChEA3 use a cutoff to limit the number of genes retained in each ChIP-seq dataset. However, TRs exhibit diverse binding patterns, with varying numbers of binding sites at different distances from TGs. Adjusting the cutoff for window size or the number of retained genes can disproportionately affect the number of identified targets for each TR. Furthermore, TRs regulate varying numbers of genes within biological processes. Maintaining a uniform number of TGs for all TRs can skew results, underrepresenting TRs that regulate many genes and overrepresenting those that regulate few. Thus, the subjective selection of cutoffs in these methods can significantly impact prediction accuracy, highlighting the need for more nuanced approaches that account for the variability in TR binding and regulatory activity.

Second, it is widely recognized that the 3D chromatin distance between an epigenomic peak and a gene promoter region, which can be measured by Hi-C or DNA-FISH [51], estimates their interaction more accurately than the one-dimensional linear distance between two loci (on a straight line) [52, 53]. However, most library-based methods use the linear distance to estimate the interactions, neglecting that the genome is organized in a complex three-dimensional structure, allowing distant locus to come in close physical contact [54, 55]. In addition, a significant fraction of binding sites has been shown to be neutral or non-functional in regulating gene expression even when located more proximal to a gene [56]. Thus, the dichotomization of genes based solely on the linear distance might lead to misleading conclusions.

Finally, library-based methods lack an approach to quantify the interaction between TRs and their TGs. Simply categorizing genes as targets and nontargets treats each TR–gene interaction as equal in importance. This neglects the fact that TRs can have different degrees of influence on gene expression. To fully understand how TRs regulate gene expression, researchers need methods that can not only identify TGs but also quantify the strength and functional significance of these interactions.

While the Fisher exact test is the primary statistical test used for enrichment analysis in library-based methods and the *P*-values generated by the test are used to assign the significance for each TR, it is important to note some variations. Methods like RegulatorTrail and TFEA.ChIP offer alternative statistical tests such as the hypergeometric test, binomial test, or gene set enrichment analysis. Additionally, several methods (Cscan, RegulatorTrail, ChEA3, and TFEA.ChIP) incorporate FDR correction approaches like the Benjamini-Hochberg method to control false positives. Notably, Enrichr discussed the potential bias in the Fisher exact test due to varying gene set sizes and addressed it by employing z-scores that account for deviations from expected ranks, which are generated through simulations.

In summary, the performance of library-based methods heavily depends on the efficiency of TG assignment algorithms. However, there is no universally accepted algorithm. The enrichment analysis can further complicate this, with different statistical methods giving different ranking lists. In addition, the limitations mentioned above can severely hinder the performance of these library-based methods.

### Region-based methods

The development of epigenomics now focuses on critical functions of CREs [57], including promoters, enhancers, silencers, and insulators. These CREs can act as the binding sites for TRs and collaborate to regulate gene transcription [58], with promoters and enhancers being the two most important ones. Therefore, unlike library-based methods which convert the TR binding sites to TGs, region-based methods avoid using a TG assignment algorithm and instead focus on the enrichment of TR binding sites in regions that contain active CREs for the input genes and use additional information to quantify the TR–gene interaction.

**Figure 4:**
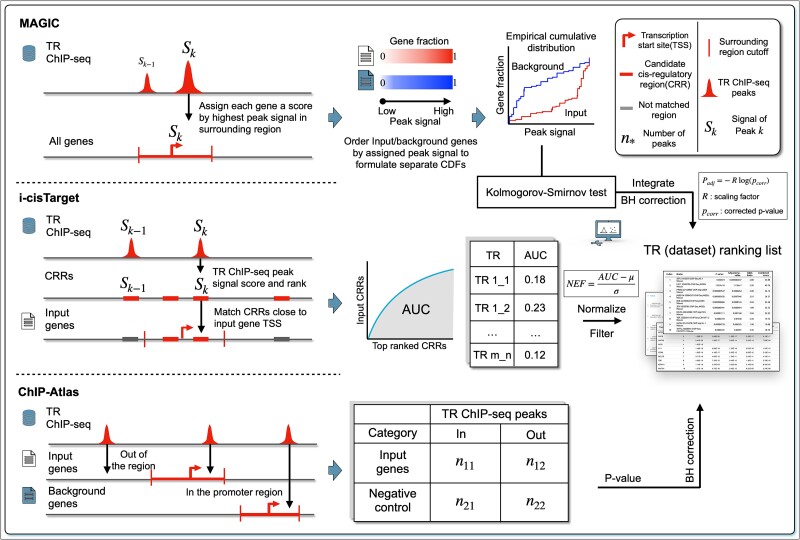
Workflow of the proximal-elements-centric method. All three methods focus on the regions that surround the gene body or gene’s TSS. TR ChIP-seq datasets with more peaks enriched in these regions would be ranked higher.

Proximal-elements-centric methods, including i-cisTarget, MAGIC, and ChIP-Atlas, mainly use proximal CREs, which are essential for basic gene transcription, to predict TRs (Fig. 4). Proximal CREs are regulatory sequences found close to the gene they regulate. Promoters, for instance, are a type of proximal CRE located immediately upstream of a gene’s TSS, which can serve as the ‘on–off’ switch to initiate gene transcription [59]. Due to their proximity to the genes that they regulate, proximal CREs often have a more direct and immediate impact on gene expression compared to distal regulatory elements located farther away from the gene locus. Past evidence suggests TRs bound to the proximal CREs usually regulate the nearby genes [60]. Therefore, the rationale behind these methods is that functional TRs should have more peaks or high-signal peaks that match the proximal CREs of their regulated genes.

i-cisTarget and MAGIC focus on peaks with high signal values. The peak signal is calculated based on the number of sequenced reads aligned to the peak position; a high signal value suggests a strong TR–DNA interaction. i-cisTarget first predefined many candidate CRRs that may contain CREs based on public sources, mainly DNaseI hypersensitive sites (DHSs). i-cisTarget scores and ranks these CRRs for each TR ChIP-seq dataset based on peak signals within the regions. Then, it calculates an area under the curve (AUC) value for each dataset by counting how many top-ranked CRRs can match the CRRs around the TSSs of input genes. Raw AUCs for the TR ChIP-seq datasets are normalized to obtain normalized enrichment scores (NEFs) and filtered by a user-selected threshold (e.g., 2.0–8.0) that yield the final ranking list of these datasets.

MAGIC assigns each gene in the input and background the highest signal value among peaks that fall into its surrounding region, and the step is repeated for each TR ChIP-seq dataset. Next, the input and background genes are ranked based on these values to create two separate empirical cumulative distribution functions (eCDFs) for each dataset. Kolmogorov–Smirnov (K-S) test is used to identify the TR ChIP-seq datasets that most effectively differentiate between the two eCDFs. The Benjamin–Hochberg corrected *P*-values from K–S tests are negative log-transformed and rescaled to generate the final scores, where the ratio of the mean of top 5% signal values assigned to the input genes versus that assigned to the background genes is used as the scaling factor.

ChIP-Atlas does not use signal values. Instead, it aims to identify which TR ChIP-seq dataset has a higher proportion of peaks enriched in the promoter regions of the input genes than in those of the background genes. Even though a one-tailed test is more appropriate here, ChIP-Atlas applies a two-tailed Fisher exact test to determine the statistical significance for each TR ChIP-seq dataset. The *P*-values generated by Fisher tests are corrected by Benjamin–Hochberg method to obtain the final ranking list.

A significant limitation of these methods lies in the unreliability of using signal strength to measure functionality. While strong peak signals often correlate with stronger binding affinity or functionality [61], they aren’t a perfect measure. This is because ChIP-seq peak signals are computed based on an average across millions of cells. Peaks with low or medium signal could represent high-affinity binding sites in a specific subpopulation of cells, while high signal peaks might reflect only moderate affinity sites present in all cells [62]. Furthermore, emerging evidence suggests that even low-affinity binding sites can play crucial roles in regulating gene transcription [63]. Therefore, a strong signal alone does not guarantee a functionally important regulatory element.

In addition to proximal CREs, distal regulatory elements like enhancers also can play crucial roles in gene transcription regulation [64, 65]. Located tens of thousands of base pairs away from their TGs, these distal CREs modulate the rate and timing of gene expression, ensuring precise control. Distal CREs are highly variable and more sensitive to environmental factors compared to proximal CREs. While incorporating distal CREs into TR prediction models could improve accuracy, directly measuring the long-range interactions between these elements and their targets remains challenging due to limited data availability.

**Figure 5:**
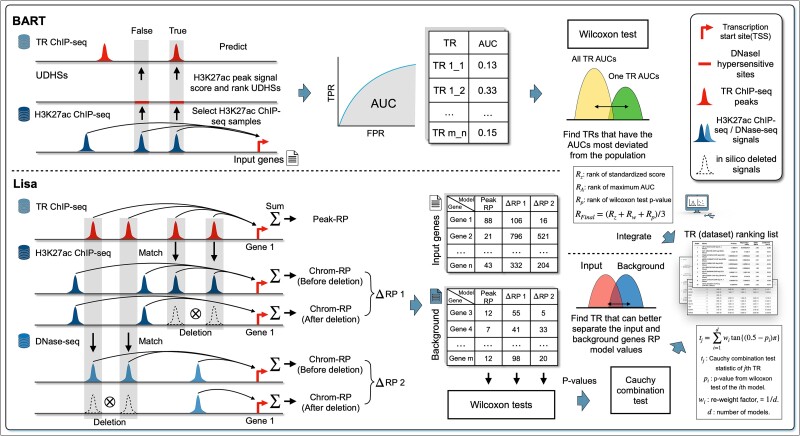
The core idea of BART and Lisa. Distal elements inclusive methods first select predictive H3K27ac ChIP-seq (or DNase-seq) samples that quantify active distal cis-regulatory elements, by summarizing the H3K27ac ChIP-seq (or DNase-seq) peak signals around a gene’s TSS. Next, statistical methods are applied to examine which TRs align more frequently with the high signal peaks from these selected samples.

The distal-elements-inclusive methods, including BART and Lisa, attempt to incorporate the activity of distal CREs by using H3K27ac ChIP-seq datasets (Fig. 5). H3K27ac is a type of Histone protein used to mark active enhancers and promoters [30]. Instead of directly identifying which TR has more peaks enriched in the proximal regions, the two methods first select a few H3K27ac ChIP-seq datasets that can be predictive for the transcription of input genes, which serve as genome-wide CREs activity landscape. The rationale of the two methods is that a functional TR should have a higher number of peaks aligned with the high signal peaks in these selected H3K27ac ChIP-seq samples.

In the selection step, a regulatory potential (RP) model is implemented in both methods, which counts the H3K27ac signals within the surrounding region of each gene to assign an RP score. Therefore, each H3K27ac dataset has a unique RP score for each individual gene. Following this, predictive H3K27ac datasets are chosen using stepwise logistic regression. That is, the two methods model the presence of a gene in the input list as a binary outcome (yes/no). The gene’s RP scores across all H3K27ac datasets are used as potential predictors. Then, using a stepwise selection method, the two methods choose a subset of these datasets that best explains whether the gene is included in the input list.

After selecting predictive H3K27ac datasets, BART collects genome-wide DHSs and for each DHS, computes a score by the weighted combination of signals from the selected datasets, and then uses these scores to predict the peak presence in each TR ChIP-seq dataset and calculates the AUC value using false positive rate and true positive rate. The AUC values of the ChIP-seq datasets are grouped by TR, and each TR’s AUC values are compared to the distribution of AUC values from all TRs through the Wilcoxon rank sum test. Finally, BART assigns each TR a unique rank by averaging the ranks of the maximum AUC value, the *P*-value from the test, and the standardized Wilcoxon statistic score that is derived using TR-specific mean and standard deviation calculated by running BART on more than 500 pre-collected gene sets.

In addition to H3K27ac datasets, Lisa selects predictive DNase-seq datasets as well; RP scores are referred to as ‘chrom-RP’ for the selected datasets of either type. After the selection, for each type, Lisa matches the peaks from each TR ChIP-seq dataset with those from the selected datasets, deleting all matched signals to reproduce the chrom-RP for each gene. Then, the fitted logistic regression model is used to calculate the chrom-RP change before and after the deletion, denoted as $\varDelta$RP for each gene. Lisa further calculates a ‘Peak-RP’ for each gene by using peaks from the TR ChIP-seq dataset. Therefore, each TR ChIP-seq dataset has two $\varDelta$RPs, one from selected H3K27ac samples and the other from selected DNase-seq samples, and one peak-RP for each gene in the input and background sets. Finally, for each TR ChIP-seq dataset, $\varDelta$RPs and peak-RPs are analyzed by three separate Wilcoxon tests to test whether there is a significant difference between the input and background, and the *P*-values are integrated by Cauchy combination test, the significance of which is used to derive a final ranking list of all TR ChIP-seq datasets.

While both BART and Lisa integrate data from other NGS techniques to analyze distal CREs, they still prioritize CREs closer to genes. Also, the presence of the H3K27ac histone mark alone might not reliably indicate CRE activity [66]. This is similar to the limitations of using peak signals from TR ChIP-seq, where a strong signal cannot guarantee functional binding.

A binding site denotes the genomic location where a transcription factor or chromatin remodeler interacts with DNA. The functionality of such a site is intrinsically linked to how the protein functions in the biological process under investigation. Since the primary role of TRs is to regulate gene expression, ideally, the quantification of binding site functionality should assess its contribution to changes in TG expression. However, current computational methods predominantly rely on proxies like epigenomic distance or binding affinity, which are not perfect measures of functional impact. The existence of long-range interactions and functional low-affinity binding sites further complicates these estimations. Without a robust quantification method, binding sites may be erroneously associated with non-TGs or their importance for TR activity may be misjudged, leading to reduced prediction accuracy. Thus, accurately quantifying the activity of CREs or the functional impact of TR binding sites on their TGs remains a critical challenge in the field.

Finally, the uneven allocation of research resources across different TRs also leads to a bias where a select few TRs and cell lines have been studied in much greater depth than others. This favoritism towards certain TRs (like CTCF and TP53) and cell lines (like HeLa and K562) often stems from research preferences or the availability of experimental materials. Since these methods rely on multiple statistical tests to assign significance to each dataset, they tend to favor TRs with extensive data coverage over those with fewer datasets.

Table 1 provides a summary of the 11 NGS-based methods examined in this study. The majority rely on data from the ENCODE [67], a trusted source for high-quality NGS data. Methods like LISA and ChIP-Atlas expand their scope by incorporating NGS datasets from individual studies. This approach offers wider TR and cell line coverage but may introduce potential variability in data quality. The evolution of these methods reflects a clear trajectory. Initially library-based, they shifted to region-based, and now employ increasingly sophisticated strategies. This highlights the field’s continuous adaptation to new NGS technologies and its ability to address increasingly complex scientific questions.

**Table 1:** The summary of evaluated eleven NGS-based methods

Method (Year)	NGS data source	Implementation	Major method to associate TFs with genes	Primary statistical method	Method categorization
Cscan (2013)	ENCODE	Web tool	Peaks fall into the surrounding fixed width window of gene TSS	Fisher exact test	Library-based
ENCODE ChIP-seq significance tool (2013)	ENCODE	NA	Peaks fall into the surrounding fixed width window of gene TSS	Hypergeometric test	Library-based
Enrichr (2013)	ENCODE/ReMap/literature	Web tool	Epigenomic distance between peaks and gene TSS	Fisher exact test/corrected FET	Library-based
i-cisTarget (2015)	ENCODE	Web tool	Peak signal to score pre-defined CRRs	Recovery analysis	Region-based
RegulatorTrail (2017)	Multiple sources	Web tool	Peaks fall into the surrounding fixed width window of gene TSS	FET/binomial test/hypergeometric test	Library-based
BART (2018)	CistromeDB	Web tool/Python	RP model to score pre-collected DHSs	Wilcoxon rank sum test	Region-based
ChIP-Atlas (2018)	NCBI / DDBJ / ENA	Web tool	Proportion of peaks fall into gene promoter region	Fisher exact test	Region-based
TFEA.ChIP (2019)	ENCODE/ReMap	Web tool/R	Peaks fall into the ‘elite’ regions from GeneHancer	FET/Kolmogorov Smirnov test / GSEA	Library-based
ChEA3 (2019)	ENCODE/ReMap/Literature	Web tool	Epigenomic distance between peaks and gene TSS	Corrected Fisher exact test	Library-based
Lisa (2020)	CistromeDB	Web tool/Python	RP model with in-silico deletion method	Wilcoxon rank sum test/Cauchy combination test	Region-based
MAGIC (2020)	ENCODE	Python	Peak signal to rank the genes	Kolmogorov–Smirnov test	Region-based

## Methods

### Benchmarking study design

We systematically compared the performance of the NGS-based methods reviewed in the previous section under a unified evaluation framework encompassing four key dimensions: accuracy, sensitivity, coverage, and usability. Also included are three motif-based methods, Pscan [15], HOMER [17], and RcisTarget [68] that can accept a gene set as input and prioritize potential TRs through motif enrichment analysis. These methods use motif libraries of known TRs and assign a higher rank to a TR whose associated motifs are determined to be over-represented. By including motif-based methods, it is possible to assess whether using NGS data can indeed enhance the prediction accuracy.

We used gene sets sourced from KnockTF, a comprehensive database of human gene expression profiles generated from TR knockdown/knockout experiments [69]. Differentially expressed (DE) genes derived from perturbation experiments often serve as a benchmark for evaluating the performance of TR-related computational methods [70]. In the database, each TR perturbation experiment has at least two control samples and two treatment samples, where Limma [71] was used to derive the DE gene set. In total, we retrieved 570 DE gene sets from the database, covering 308 known TRs.

Input gene sets, typically derived from differential or correlated gene expression analysis, often contain genes with varying significance or correlation levels. Researchers must then subjectively select a subset of genes based on these values, which can be an arbitrary process. However, including too many genes can introduce noise from less significant or irrelevant genes, potentially obscuring the true regulatory relationships and negatively impacting prediction accuracy. To investigate the effect of input gene set size on TR identification performance, we selected the top 200, 600, and 1000 most significant genes from each of the 570 DE gene sets. We used the top 200 genes to evaluate accuracy, as these are expected to be most strongly associated with the perturbed TR. The larger gene sets (600 and 1000) were used to assess method sensitivity to noise, i.e., how well the methods perform when less relevant genes are included.

Annotated genes were converted to the required format as per each method’s manual or instructions. Default settings were used whenever available, otherwise, parameters were chosen based on the provided documentation. It is worth mentioning that besides the input gene set, most methods also use a background gene set, typically comprising all annotated or non-significant genes, as a reference for statistical comparison. While methods like Cscan, ChEA3, MAGIC, and RegulatorTrail use a fixed background of all annotated genes, others like Enrichr, TFEA.ChIP, ChIP-Atlas, and Lisa allow user customization. BART and i-cisTarget bypass the need for a background set due to their unique methodologies. In our evaluation, we employed the default setting of the background set (all annotated genes) for the four customizable methods.

To assess a method’s accuracy, we focused on its ability to rank known, perturbed TRs at the top of its generated ranking list. This aligns with the user’s primary interest: identifying the most relevant TRs at the forefront of the results. We first selected a range of threshold values ($K$) for “top $K$ positions” ($K$=10, 50, 100). For each $K$ value, we then applied four widely recognized metrics designed for evaluating ranking algorithms to assess the ranking results from the different methods [72]. Given gene lists $i=1,2,\dots, N$, $N$ is the total number of evaluated gene lists. The terms ranked above threshold are ordered as $k=1,2,\dots, K$ by their ranks. The relevance of each term in the ranking list is then denoted as ${R}_{ik}$, where ${R}_{ik}=1$ if the term is indeed the perturbed TR, and 0 otherwise. The total number of relevant terms in the ranking list $i$ is denoted as ${n}_{iK}$, where ${n}_{iK}={\sum}_{k=1}^K{R}_{ik}$ does not exceed the number of TR ChIP-seq data sets associated with the perturbed TR. The metrics used in our evaluation include (1) Hit rate at $K$, which calculates the proportion of ranking lists that contain at least one relevant term above the threshold,


$$ {H}_K=\frac{1}{N}\sum_{i=1}^NI\left\{{n}_{iK}>0\right\}. $$

(2) Mean reciprocal rank (MRR) at $K$, which values the first relevant term in each ranking list,


$$ MR{R}_K=\frac{1}{N}\sum_{i=1}^N\frac{1}{k_i^{\ast }},{k}_i^{\ast }={\mathrm{argmin}}_k\left({R}_{ik}=1\right). $$

(3) Mean average precision (MAP) at $K$, which measures a method’s capability of ranking relevant terms at the top, penalizing deviations. For a ranking list $i$, the metric first calculates the precision ${P}_i(k)$ at every $k$. It then sums over all $k$, further divided by ${n}_{iK}$ for list $i$. Finally, it averages across all the ranking lists to get the overall precision.


$$ MA{P}_K=\frac{1}{N}\sum_{i=1}^N\left[\frac{1}{n_{iK}}\sum_{k=1}^K{P}_i(k)\right],{P}_i(k)=\left\{\ \begin{array}{l}\sum_{j=0}^{k-1}\frac{R_{i,k-j}}{k},\kern0.75em \mathrm{if}\ {R}_{ik}=1.\\{}0,\kern0.75em \mathrm{otherwise}.\end{array}\right. $$

(4) Mean normalized discounted cumulative gain (mNDCG) at $K$, which also considers the position of relevant terms in a ranking list while giving more weights to the terms placed higher.


$$ mNDC{G}_K=\frac{1}{N}\sum_{i=1}^N\left[\sum_{k=1}^K\frac{R_{ik}}{\log_2\left(k+1\right)}/\sum_{k=1}^{n_{iK}}\frac{1}{\log_2\left(k+1\right)}\ \right]. $$

Each of the four metrics has a different focus. The hit rate measures in general whether a method can identify the perturbed TRs. While MRR, MAP, and mNDCG consider the relevant positions of perturbed TRs in the top, MRR focuses exclusively on the ranking of the first relevant term. In case a method ranks multiple datasets linked to the same TR above the threshold, MAP and mNDCG evaluate the rankings of all relevant terms but assign different weights and penalties. In addition to the ranking quality metrics that consider the absolute positions of perturbed TRs, we also counted the number of ranking lists that have perturbed TRs shown in the top 1%, top 5%, and top 10%. The accuracy of each method is ranked by their average performance over all metrics and thresholds.

We also evaluated the sensitivity of methods to noisy input, focusing on their ability to produce consistent and reliable results. We tested methods with $G=200,600,1000$ genes that are most significant, respectively. The size of the input gene set is the major parameter that can be controlled by users. As $G$ increases, the number of non-significant genes in the gene set tends to increase, which may negatively affect the performance. In addition, we investigated whether TRs with multiple ChIP-seq datasets are significantly ranked higher compared to TRs with only one dataset by using Wilcoxon test. The *P*-values derived from gene sets of the same size are integrated by Stouffer’s Z-score method. Let $i$ indicate the $i$th ranking list; ${p}_i$ is the *P*-value of Wilcoxon test by comparing the rankings of TRs with multiple ChIP-seq datasets with TRs with only one dataset in the $i$th ranking list; ${Z}_i$ and ${Z}_c$ are individual and combined Z-scores computed by the Stouffer’s method; ${p}_c$ is the final combined *P*-value.


$$ {Z}_i={\Phi}^{-1}\left(1-{p}_i\right),{Z}_c=\frac{\sum_{i=1}^I{Z}_i}{\surd I},{p}_c=1-\Phi \left({Z}_c\right) $$

Beyond accuracy and sensitivity, a method’s coverage is crucial. This refers to the breadth of TRs and cell lines represented in its internal NGS database. Without relevant data, a method cannot rank a specific TR, excluding it from results. Therefore, coverage directly impacts performance—broader coverage allows for wider analysis and a greater chance of identifying relevant TRs. Methods with continually updated databases tend to have better coverage, reflecting the latest discoveries.

Even high-performing methods can face practical implementation challenges. Therefore, usability, determined by factors like ease of implementation, documentation quality, and code clarity, is critical. To assess usability, we defined twelve binary criteria (0 = not met, 1 = met) addressing key user considerations when selecting a method: (i) a manual of clear instructions about method usage, (ii) illustrative examples helping users understand the analysis workflow, (iii) output explanation helping users interpret method results, (iv) availability of a CLI/API or R/Python package facilitating large-scale analyses, (v) a user-friendly web tool allowing direct method execution, (vi) easy implementation without the need for extensive customized coding, (vii) open-source code allowing for transparency and further development options, (viii) clear code annotations facilitating user comprehension of the program structure and logic, (ix) active maintenance ensuring the method remains functional, (x) visualization enhancing user comprehension of analysis results, (xi) data import accepting gene symbols without requiring complex conversions, and (xii) minimal unexpected errors during use ensuring a smooth user experience.

Within this evaluation framework, we benchmarked thirteen methods. Note that we excluded the ENCODE ChIP-seq significance tool due to being outdated and inaccessible. For Enrichr, we used the highest ranks from four libraries. For ChEA3, we followed the original publication’s recommended approach of utilizing the integrated mean rank. Additionally, it’s important to note that i-cisTarget web tool employs a filtering step, therefore only datasets that pass this filter were included in our evaluation.

## Results

### Accuracy

**Figure 6:**
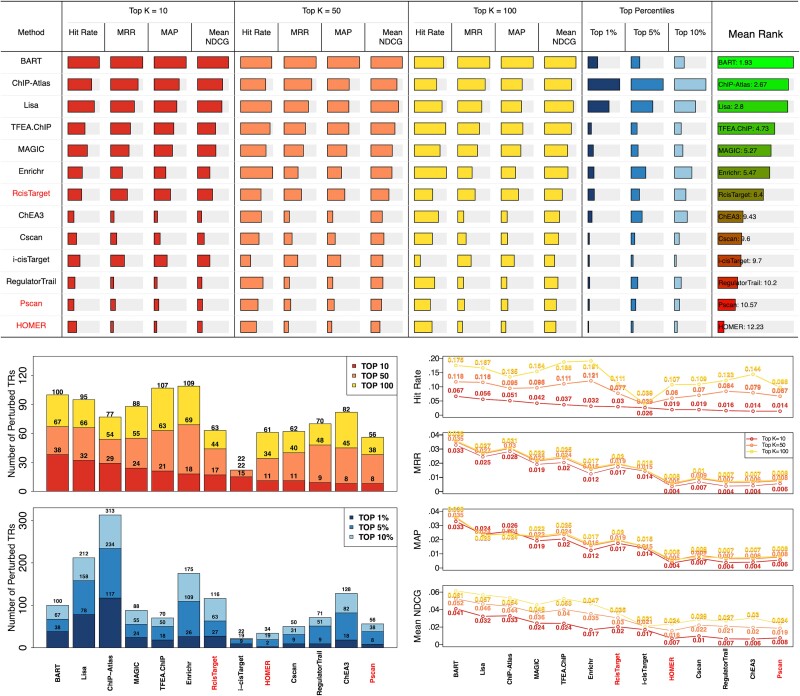
Comparison of accuracy. RcisTarget, Pscan and HOMER are motif-based methods. BART, ChIP-Atlas, and Lisa demonstrate the best overall performance across different scenarios. The NGS-based methods generally outperform the motif-based methods. All methods ranked fewer than 10% of perturbed TRs within the top 10 positions, suggesting a room for further improvement.

Figure 6 provides an overview of the performance of thirteen methods evaluated for ranking perturbed TRs. In summary, no single method stood out as the best in all evaluation metrics. However, actively updated or recently developed methods were observed to perform better, perhaps due to their larger accumulation of NGS data and more sophisticated algorithm design. Some general trends and potential issues were also observed, as detailed below.

First, BART, ChIP-Atlas, and Lisa are recommended in general as they are the top three performing methods in most scenarios. For example, BART, ChIP-Atlas, and Lisa have higher hit rate, MRR, MAP, and NDCG values for three selected thresholds. They also performed well by considering the top percentile rankings. The good performance is partially credited to the extensive collections of NGS data: ChIP-Atlas contains over 30 000 human TR ChIP-seq datasets, BART and Lisa have around 7000 human datasets, while other methods only have 1000 to 3000 datasets. Furthermore, the region-based approach might also play a pivotal role in enhancing their accuracy. TFEA.ChIP, MAGIC, Enrichr, and RcisTarget also show good performance under certain metrics. Besides, NGS-based methods outperform motif-based methods on average. No motif-based method evaluated can surpass the performance of the top-performing NGS-based methods (e.g., BART, ChIP-Atlas, and Lisa), regardless of the metrics and thresholds used.

**Figure 7:**
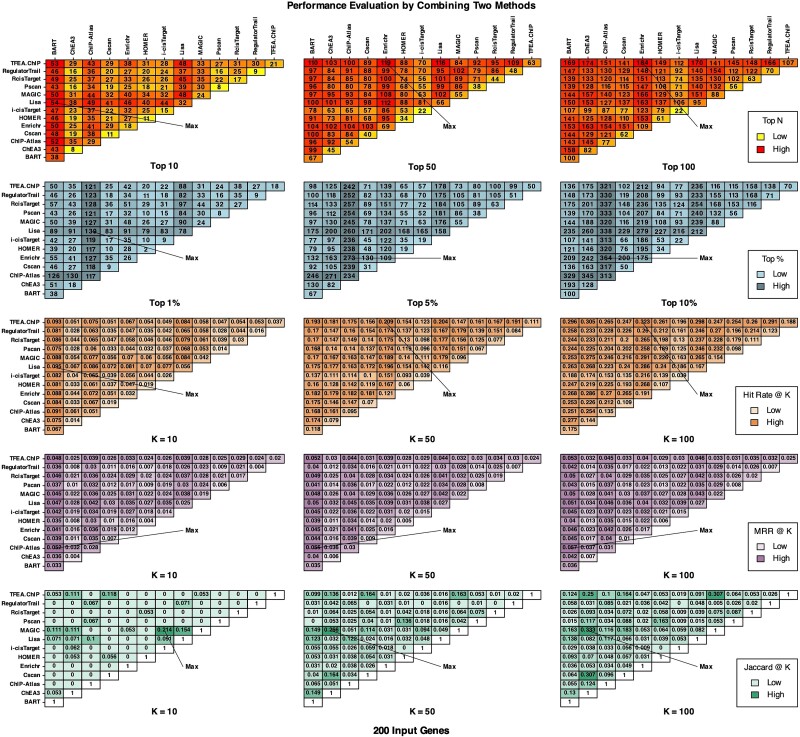
The number of perturbed TRs ranked to top positions by either method in the combination of two methods, with MRR and hit rate calculated by using the highest rank of the relevant term in the combined output list, and the Jaccard index to measure the similarity of top terms (200 input genes used). The combination of any two from BART, ChIP-atlas, Lisa, Enrichr, and TFEA.ChIP can achieve the best or close to the best performance.

Combining results from various methods is believed to be more effective than relying on a single method due to differences in databases and algorithms used. We next explore combining two methods to determine which pairs give the best results among all. Figure 7 illustrates the number of perturbed TRs that are ranked in the top 10, 50, and 100 and the top 1%, 5%, and 10% in at least one TR ranking list when two methods are used (200 input genes in this figure; see Figures S1 and S2 for 600 and 1000 input genes). We also calculated the MRR and hit rate by using the highest rank of the relevant term in the combined output list of two methods and examined the similarities between the top 10, 50, and 100 terms of any two methods by using Jaccard index. Figure 7 shows that overall, Lisa, TFEA.ChIP, BART, and ChIP-Atlas have potential for complementary use, as their distinct ranking profiles, as indicated by lower Jaccard, may capture a wider range of perturbed TRs when combined.

**Figure 8:**
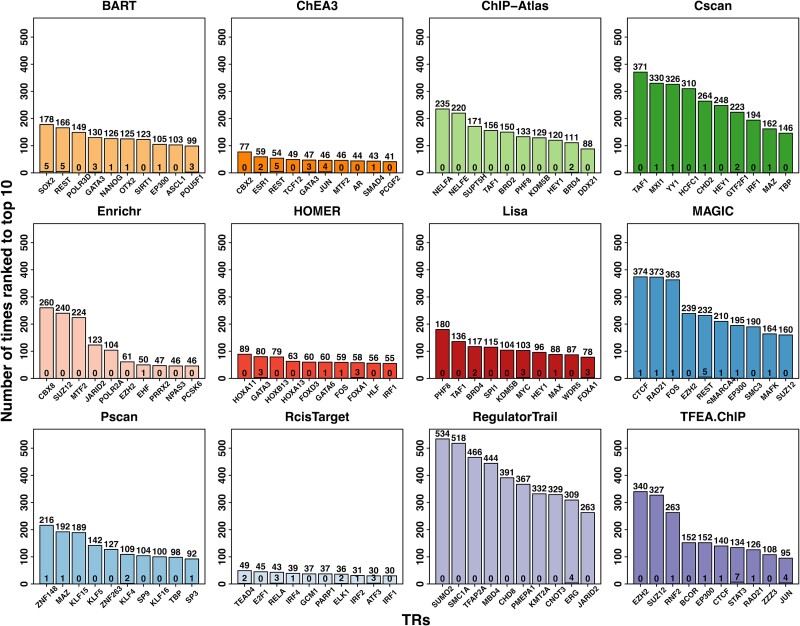
TRs that are most frequently ranked to the top 10 positions by different methods. The number at the top of each bar indicates how many times the corresponding TR is ranked in the top 10 and that at the bottom indicates how many times that it is indeed the perturbed TR (out of 570). i-cisTarget was excluded as the number of TRs in the final ranking list is often less than 10 due to the threshold.

Figure 8 reveals a tendency among many methods to consistently rank certain TRs highly, regardless of the input gene set. However, these ‘favorite’ TRs are rarely the experimentally perturbed TRs we want to identify. Prioritizing irrelevant TRs can overshadow true functional regulators, pushing them lower in the ranking list. This issue is most pronounced in RegulatorTrail, Cscan, and MAGIC. Thus, even if top-ranked TRs seem expected or familiar, we recommend users cross-check them against a method’s ‘favorite’ TR list before proceeding with further analysis.

### Sensitivity

**Figure 9:**
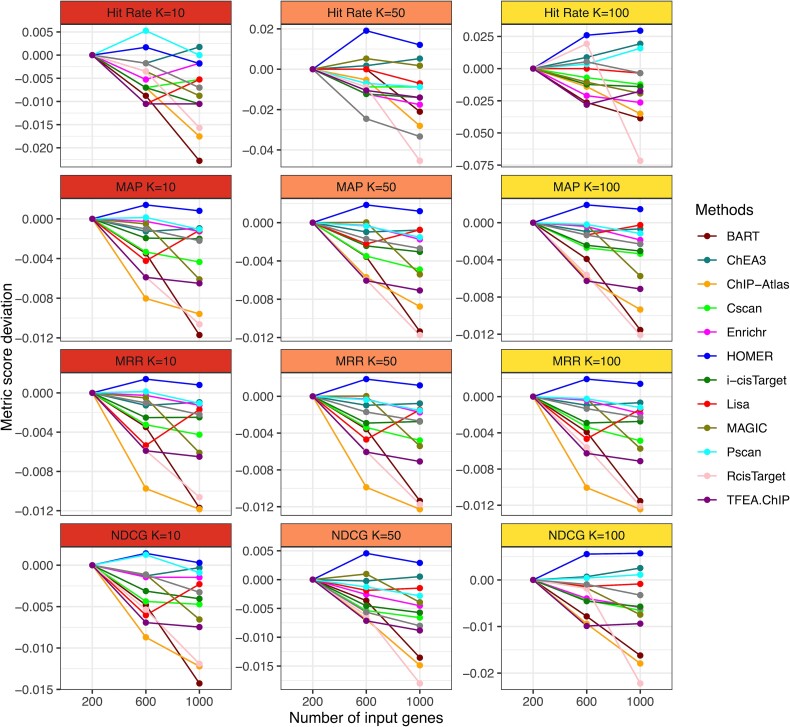
As the size of the input gene set increases, the majority of methods demonstrate a decline in performance. Notably, HOMER, Pscan, and ChEA3 show relatively stable or even better performance with increased number of input genes. For the top three performing methods in the evaluation of accuracy, Lisa displays the most consistent performance compared to BART and ChIP-Atlas.

Figure 9 presents results from the sensitivity analysis with three input gene set sizes. As the input size increases, most methods have reduced performance as we expect, indicating that the inclusion of more genes into the input set will lead to decreased performance of most methods. Therefore, one should be cautious when selecting genes used as input for these methods. The number of input genes itself can be a factor affecting the method performance. It is noted that HOMER, Pscan, and ChEA3 show relatively stable performance compared to others. However, given that accuracy remains the major consideration, among the top-performing methods (e.g., Lisa, ChIP-Atlas, and BART), Lisa is less sensitive to the change of input size.

Except for the input gene set size, another major concern is the uneven allocation of research resources, as some TRs have been extensively investigated (Fig. S3). We selected 20 TRs with the largest numbers of ChIP-seq datasets and 20 TRs associated with just one dataset randomly from the ENCODE database. For each gene set, we employed a one-sided Wilcoxon rank-sum test to determine if the 20 TRs with multiple datasets tend to be ranked higher than those with a single dataset. Additionally, we excluded gene sets that feature selected TRs as the perturbation targets, ensuring that each gene set could be considered a randomly generated set that is irrelevant to any of the selected TRs. The findings, detailed in Table S1, reveal that most methods indeed tend to favor TRs associated with a larger number of datasets. ChEA3 is the only method that does not give significant results, which might be due to the integration step of using mean ranks of all libraries as the final rank. This bias might explain why certain TRs consistently appear at the top as shown in Fig. 8.

Finally, we illustrated how the value of a cutoff can affect the rankings of individual perturbed TRs that are expected to be ranked high by any appropriate methods (Fig. S4–S5). We sourced ~2000 TR ChIP-seq datasets from the ENCODE project and implemented similar workflows as Cscan and Enrichr, both library-based methods, to identify TGs, followed by a one-sided Fisher exact test for each TR to produce the rank based on its significance. To assign TGs, Cscan uses windows of a predetermined width and Enrichr retains a prefixed number of genes based on the genomic distance, as discussed before. The figures show that, no matter which quantity is used as the cutoff, the individual ranks of some perturbed TRs can change drastically as we vary the cutoff, which again confirms that the use of a hard cutoff may pose a challenge in these methods.

### Coverage

**Figure 10:**
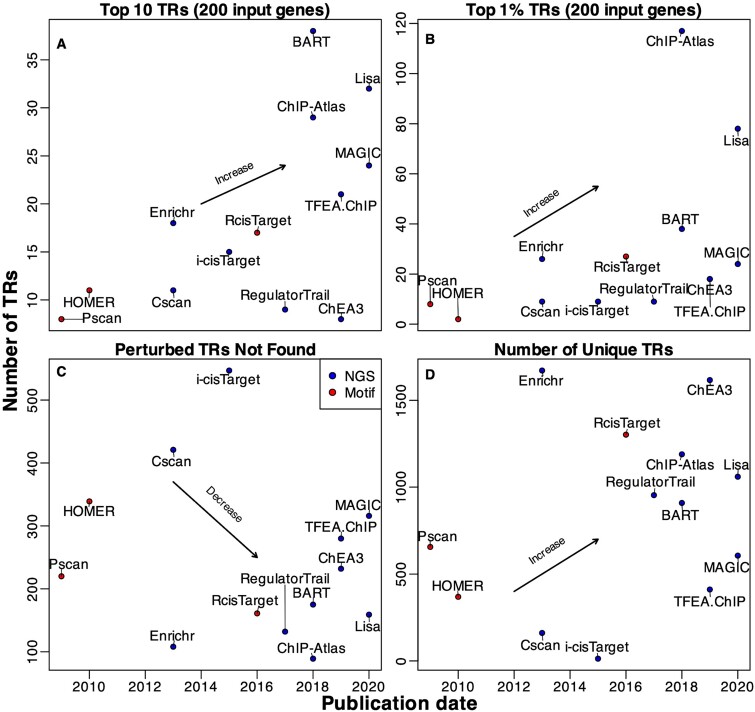
An increasing trend in prediction performance and TR coverage, aligning with more recent publication dates. (A) No. of perturbed TRs ranked within the top 10 positions versus method’s publication date; (B) No. of perturbed TRs ranked within top 1% versus method’s publication date; (C) No. of perturbed TRs that cannot be found in each method’s TR ranking list versus its publication date; (D) No. of unique TRs in the TR ranking list of each method versus its publication date.

With the detailed analysis of accuracy, it is evident that coverage plays a crucial role in the effectiveness of methods. The extent to which these methods can incorporate up-to-date NGS data can be a significant factor contributing to the performance (Fig. 10). First, Fig. 10A and B show that the publication time has a positive correlation with the ranking performance and TR coverage, which confirms that the continuing efforts from generations of researchers have improved the prediction performance of algorithms to address the problem. Second, Fig. 10C displays the number of perturbed TRs that are missing in the TR ranking list versus the publication date for each method. As we expect, more recently developed methods generally have lower numbers of missing TRs. Figure 10D plots the number of unique terms displayed in the TR ranking list of each method. Considering that the benchmark gene sets used in this study only cover a limited number of known TRs, a high number of missing TRs coupled with a low number of available terms might suggest an insufficient coverage of TRs. Notably, ChIP-Atlas, Lisa, and BART demonstrated their relatively comprehensive coverage of NGS data, which resonates with their better accuracy observed before, indicating the method performance is positively correlated with the number of covered TRs in method’s database.

### Usability

**Figure 11:**
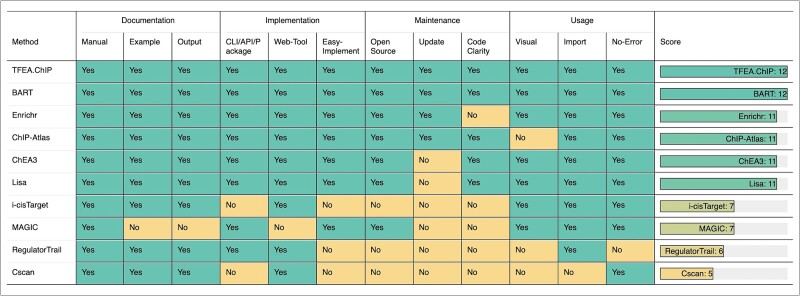
The summary of usability evaluated by using nine criteria covering most user considerations for method usage. ‘Yes’ indicates the method satisfied the criterion and a ‘No’ otherwise. Enrichr, TFEA.ChIP, BART, ChIP-Atlas, ChEA3, and Lisa received good scores.

Figure 11 provides an assessment of NGS-based methods using various criteria of usability, with a ‘Yes’ indicating that the criterion is satisfied and a ‘No’ otherwise. In general, Enrichr, TFEA.ChIP, BART, ChIP-Atlas, ChEA3, and Lisa received good scores as these methods are generally user-friendly, easy to learn and use, and did not pose any issues during testing. In summary, we recommend refraining from using methods that are not open-source, actively updated, or well-maintained. Such methods often rely on outdated databases and could pose compatibility issues.

## Conclusion

Our evaluation of NGS-based methods in four dimensions leads us to recommend Lisa, ChIP-Atlas, and BART, either individually or in combination, for predicting TRs of query gene sets. These methods have demonstrated relatively better performance in providing accurate predictions with perturbed TR gene sets. The evaluated methods have three major limitations in general: (i) Peaks are associated with genes based on linear epigenomic distance. (ii) There is no ideal approach to quantify the functionality or activity of binding sites or CREs. (iii) The internal database of reviewed methods can have a highly skewed distribution of the number of datasets for each TR. In addition, it is important to note that even the best-performing method may only rank less than 10% of perturbed TRs within the top 10 positions. Given the complexity of cellular processes and the limitations above, researchers should use caution when interpreting the results and should consider further validation with experimental approaches. Nevertheless, recently developed NGS-based methods provide useful tools for generating hypotheses about transcriptional regulation. Further methodological research is needed to fully utilize the wealth of NGS data and address the limitations of current methods.

## Discussion

This study evaluates various computational methods for predicting TRs from query gene sets, highlighting both the potential and challenges inherent in NGS-based approaches. While NGS technologies provide unprecedented access to context-specific TR binding profiles, their accuracy is affected by the complexity of cellular biology and technical biases in the sequencing process.

### Biological complexities of TR regulation

TR binding is inherently dynamic, varying across cell types and time in response to diverse signals and environmental factors. TRs often bind to different DNA regions in different cell types, and their binding patterns can change over time depending on cellular conditions and external stimuli [73]. Chromatin accessibility and epigenetic modifications, such as DNA methylation and histone modifications, play a crucial role in modulating TR binding, as these factors can influence the availability and affinity of binding sites [74, 75]. Additionally, TRs frequently cooperate with other proteins, forming complexes that can alter their binding specificity and affinity [76]. This intricate interplay between TRs, DNA, and other cellular components creates a complex landscape of interactions that challenge accurate prediction and functional interpretation.

The evaluated computational methods employ various strategies to mitigate these challenges. For example, region-based methods such as Lisa and BART integrate TR binding profiles with epigenetic modification and chromatin accessibility profiles. This combination helps correlate binding sites with histone modification sites and accessible regions, facilitating the investigation of regulatory mechanisms in cell lines that lack TR ChIP-seq experiments. Methods like TFEA.ChIP leverage existing GeneHancer database to identify long-range interaction between TR and TGs. Crucially, all methods strive to build comprehensive reference databases by incorporating NGS experiments across multiple cell types, aiming to capture the inherent diversity in TR binding profiles.

### Sequencing biases in NGS-based TR prediction

The reference NGS data utilized by these methods are often compiled from diverse sources, including public consortia and individual publications, potentially originating from different laboratories and employing various sequencing technologies. This heterogeneity can introduce platform-specific biases that affect the accuracy of TR analyses.

These biases manifest in various ways [77]. For example, Illumina platforms are known to exhibit a strong GC bias, wherein genomic regions with high or low GC content may be over- or under-represented in the sequencing data [78]. This can lead to erroneous identification of TR binding sites, particularly in regions with extreme GC content. In contrast, newer long-read sequencing technologies like PacBio and Oxford Nanopore, while less prone to GC bias, can introduce other biases related to their unique sequencing chemistries, such as systematic errors in homopolymer regions [79]. Furthermore, biases introduced during library preparation, such as PCR amplification bias [80], can also significantly impact the accuracy of NGS-based methods. Preferential amplification of certain sequences can lead to overrepresentation of specific genomic regions, resulting in the false identification of TR binding sites or the masking of true binding events. These platform-specific biases can confound the interpretation of NGS-based results, particularly when reference datasets are compiled from diverse sources. Therefore, understanding the inherent limitations of different sequencing technologies and carefully considering their potential biases is crucial for the accurate and reliable TR prediction using NGS-based methods.

### Future directions and opportunities

Despite current advancements, new approaches are needed to address remaining limitations. Previous studies have implicated several possible directions. First, further processing of the NGS data by using ‘foot printing’ methods to identify binding sites in chromatin accessibility data was reported before [81]. However, this approach is limited to TRs with long residence times on chromatin, despite some TRs being known for lack of detectable footprints [82, 83]. Second, including three-dimensional long-range interaction information from Hi-C data may also serve as a possible improvement direction [84–86], but the sparse nature and low resolution of Hi-C data might also cause misleading conclusions and therefore should be used with caution and further validation. Third, the inclusion of additional NGS data, for example, DNA methylation data, can help elucidating the varying context-specific binding profile caused by epigenetic modification.

It is also noted that the development of deep learning algorithms has broad applications in transcription regulation research. Although most deep learning methods currently focus on tasks such as predicting transcription factor binding sites [87–89], reconstructing GRNs [90, 91], deconvoluting bulk NGS data to obtain cell-type level information [92], or using protein sequence information to predict transcription factors [93].

Deep learning algorithms also offer significant potential. While current applications focus on predicting sites [87–89], reconstructing GRNs [90, 91], deconvoluting bulk NGS data [92], and predicting TFs from protein sequences [93], their integration with existing methods could enhance performance. For example, deep learning predicted TFBSs can be used as context-specific binding profiles in certain biological processes that have not been measured by any NGS experiment.

The rise of single-cell sequencing technologies (scRNA-seq, scATAC-seq, scChIP-seq [94]) presents exciting opportunities. By providing granular, cell-level resolution, these techniques can overcome limitations of bulk sequencing data, such as inaccurate binding affinity measurements due to averaging across heterogeneous cell populations [95]. In addition, with the rise of multi-omics experimental approaches [96, 97], the availability of multi-omics data is no longer restricted to one or two types, and there is a growing need for novel multi-omics approaches that can leverage the full range of available data.

In the coming decade, we anticipate a surge in both single-cell and multi-omics approaches for predicting functional TRs. These advancements will drive innovation and discovery in the field of transcriptional regulation, leading to a deeper understanding of the complex interplay between TRs and cellular processes.

**Key Points**

- An introduction to available computational methods for predicting functional TRs from a query gene set.
- A detailed walk-through along with practical concerns and limitations.
- A systematic benchmark of NGS-based methods in terms of accuracy, sensitivity, coverage, and usability, using 570 TR perturbation-derived gene sets.
- NGS-based methods outperform motif-based methods. Among NGS methods, those utilizing larger databases and adopting region-centric approaches demonstrate favorable performance. BART, ChIP-Atlas, and Lisa are recommended as these methods have overall better performance in evaluated scenarios.

## Supplementary Material

Supplementary_Material_bbae366

## Data Availability

We established a permanent data repository on Zenodo (https://zenodo.org/records/11391962), which includes the TR perturbed gene sets, ranking lists generated by each method, as well as commands and scripts. TR perturbation derived DEGs are sourced from KnockTF [69] (https://bio.liclab.net/KnockTFv1/). ChIP-seq data are sourced from ENCODE [67] (https://www.encodeproject.org/).
